# Echocardiographic evidence of an intrapulmonary shunt in a patient with severe liver cirrhosis

**DOI:** 10.1007/s00392-021-01817-y

**Published:** 2021-02-23

**Authors:** Henrike Dobbermann, Christoph Marquetand, Jens U. Marquardt, Jan-Christian Reil

**Affiliations:** 1 Klinik Für Innere Medizin I, Universitätsklinikum Schleswig-Holstein, Campus Lübeck, Ratzeburger Allee 160, Lübeck, Germany; 2 Klinik Für Innere Medizin II, Kardiologie, Angiologie Und Internistische Intensivmedizin, Universitäres Herzzentrum Lübeck, Universitätsklinikum Schleswig-Holstein, Campus Lübeck, Ratzeburger Allee 160, Lübeck, Germany

**Keywords:** Echocardiography, Hepatopulmonary syndrome, Shunt, Contrast medium, Dyspnea

Sirs:

Echocardiography is the most useful screening test [1] in diagnosing intrapulmonary shunts in patients with severe liver cirrhosis and those suspected of having hepatopulmonary syndrome.

The provided image (see Fig. 1) is an anatomic M-mode representation of the left and right ventricles of a patient who suffered from gradual progressive shortness of breath in the previous weeks and cryptogenic liver cirrhosis. Exactly three heart beats after having reached the right ventricle the contrast medium, a Gelafundin^Ⓡ^ air mixture that normally cannot pass the pulmonary vasculature, opacifies the left heart chambers. The corresponding video demonstrates the influx of contrast medium through the pulmonary veins (see video 1 and Fig. 2). This finding is highly suggestive for intrapulmonary shunts. Accordingly, ASD and PFO were excluded by TOE examination 5 years before. The further echocardiographic examination revealed normal left and right ventricular function and excluded valvular heart disease. Additionally, the echo-derived pulmonary vascular resistance was rather low (PVR Echo = 0.1618 + 10.006 * TRV_max_/VTI_RVOT_ according to [2]  = 0.1618 + 10.006*1.6 m/s  / 18 cm = 1.05 = 1.05 Wood Units) and cardiac output was slightly increased up to 6.5 L/min while right and left ventricular flow ratio Qp/Qs was about 1, thereby further indicating lack of intracardiac shunts. The patient showed severe hypoxemia with a PO_2_ of 40 mmHg in ambient air, while dyspnea symptoms improved subjectively when the patient laid down (platipnoea). The triad of liver disease with hypoxemia caused by intrapulmonary shunts is characteristic for the hepatopulmonary syndrome. Pathophysiologically, the impaired liver function possibly favours the accumulation of vasoactive substances in the lung thereby reopening small vascular shunts [3]. Hepatopulmonary syndrome is estimated to be found in 4–47% of patients with liver disease [4]. The prognosis of these patients is rather limited with a median survival of 10 months [5]. Liver transplantation is currently the only existing therapy in this situation [6]. After diagnosis of hepatopulmonary syndrome our patient was listed for high urgency liver transplantation, but unfortunatedly died 4 weeks later. Considering the clinical implications, patients with HPS and liver disease who are suffering from dyspnea should be tested for intrapulmonary shunts to accelerate the evaluation process for liver transplantation as early as possible..

**Fig. 1:**
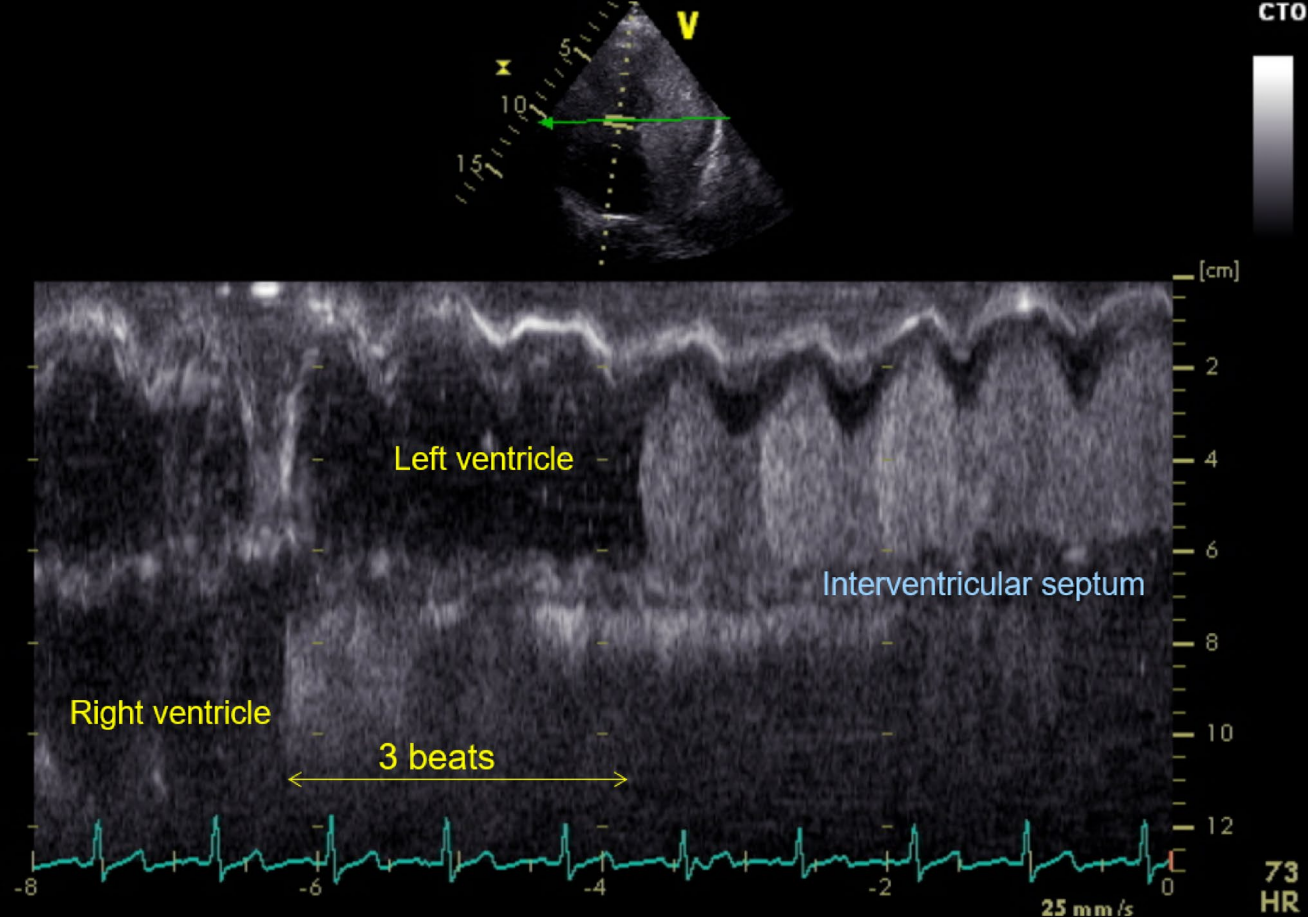
Anatomical M-mode of the left and right ventricle. The left ventricle is opacificated by contrast medium (Gelafundin^®^ air mixture) three heart cycles after the right ventricle

**Fig. 2:**
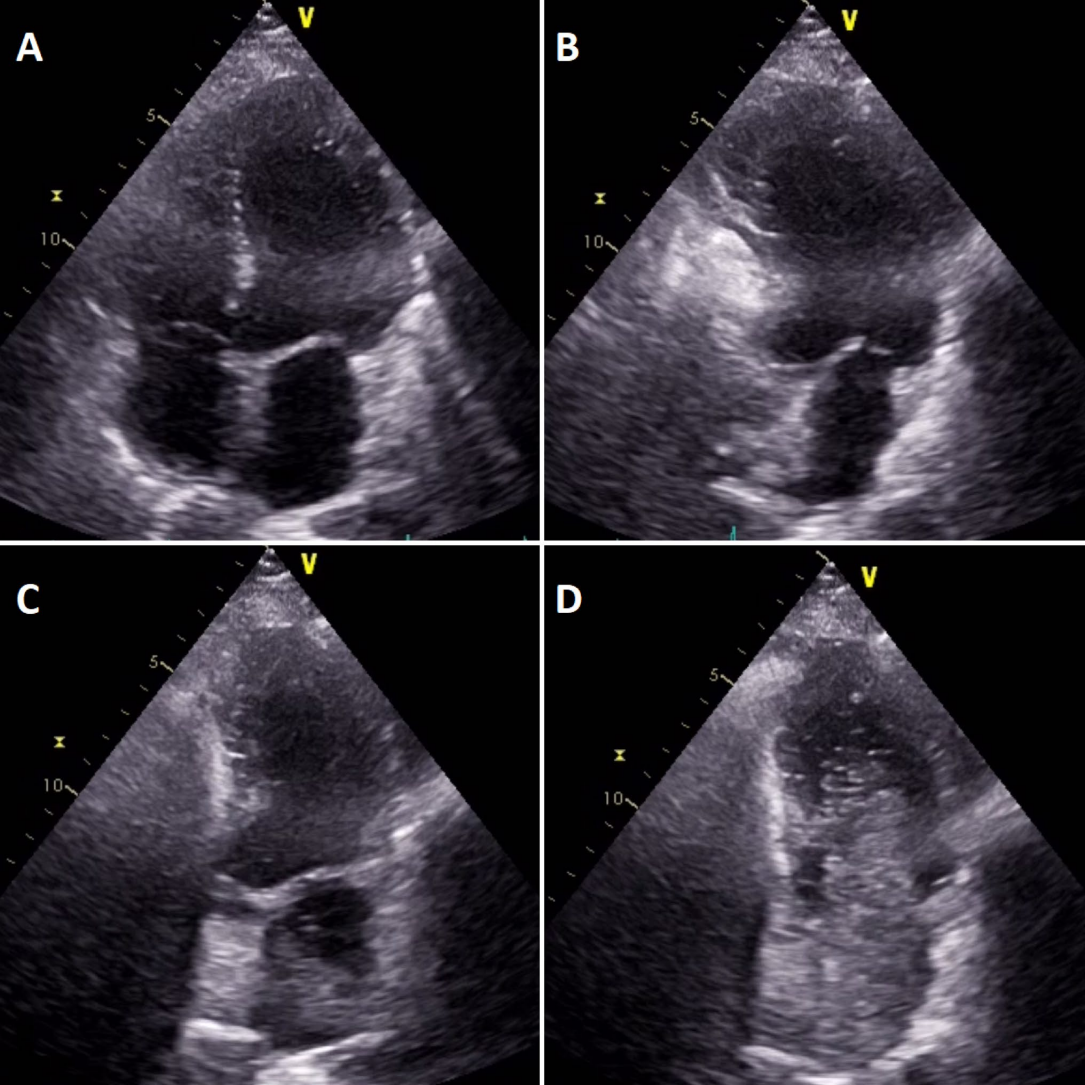
Still view of video 1. Panel **a** shows the 4 chamber view of the heart without contrast medium. Panel **b** shows opacification of the right chambers. Panel **c** shows the contast medium shows the influx of the contrast medium through the pulmonary veins into the left atrium. Panel **d** shows the opacification of the left ventricle

## Supplementary Information

Below is the link to the electronic supplementary material.

Supplemental Video 1: Apical five chamber view after application of an intravenous bolus of contrast medium (Gelafundin^®^ air mixture) (avi 6882 KB)
